# A secretory phospholipase A_2_-mediated neuroprotection and anti-apoptosis

**DOI:** 10.1186/1471-2202-10-120

**Published:** 2009-09-23

**Authors:** Arunmozhiarasi Armugam, Charmian DN Cher, KaiYing Lim, Dawn CI Koh, David W Howells, Kandiah Jeyaseelan

**Affiliations:** 1 Department of Biochemistry, Yong Loo Lin School of Medicine, National University of Singapore, 8 Medical Drive, 117597, Singapore; 2 National Stroke Research Institute, Austin Health, Studley Rd, Heidelberg, Victoria 3084, Australia

## Abstract

**Background:**

Phospholipase A_2 _liberates free fatty acids and lysophospholipids upon hydrolysis of phospholipids and these products are often associated with detrimental effects such as inflammation and cerebral ischemia. The neuroprotective effect of neutral phospholipase from snake venom has been investigated.

**Results:**

A neutral anticoagulant secretory phospholipase A_2 _(nPLA) from the venom of *Naja sputatrix *(Malayan spitting cobra) has been found to reduce infarct volume in rats subjected to focal transient cerebral ischemia and to alleviate the neuronal damage in organotypic hippocampal slices subjected to oxygen-glucose deprivation (OGD). Real-time PCR based gene expression analysis showed that anti-apoptotic and pro-survival genes have been up-regulated in both *in vivo *and *in vitro *models. Staurosporine or OGD mediated apoptotic cell death in astrocytoma cells has also been found to be reduced by nPLA with a corresponding reduction in caspase 3 activity.

**Conclusion:**

We have found that a secretory phospholipase (nPLA) purified from snake venom could reduce infarct volume in rodent stroke model. nPLA, has also been found to reduce neuronal cell death, apoptosis and promote cell survival in vitro ischemic conditions. In all conditions, the protective effects could be seen at sub-lethal concentrations of the protein.

## Background

Phospholipase A_2 _(PLA_2_; EC 3.1.1.4) forms a diverse class of enzymes with regard to structure, function, localization and regulation. The enzyme catalyzes the hydrolysis of the *sn*-2 fatty-acyl bond of phospholipids to liberate free fatty acids and lysophospholipids [1]. Major groups of phospholipase A_2 _that have been actively studied in mammalian systems include a) cytosolic Ca^2+^-dependent (cPLA_2_) or Ca^2+^-independent (iPLA_2_) [2,3] and b) Ca^2+^-dependent secretory (sPLA_2_) phospholipase A_2_. Both the cPLA_2 _and iPLA_2 _are high molecular weight (85-110 kDa) intracellular enzymes [4] and they have been widely associated with multifaceted network of signaling pathways. However, PLA_2 _that acts on membrane phospholipids has been implicated in cell death and differentiation as well as intracellular membrane trafficking [5].

In mammalian cells, PLA_2 _activity has been found to increase in response to numerous stimuli such as osmotic challenge, oxidative stress, ischemic conditions and exposure to allergens. The synthesis of different cell specific sub-types and activation of mammalian PLA_2 _are associated with cell injury and various pathophysiological conditions [6]. In the central nervous system (CNS), PLA_2 _is known to participate in many (patho)physiological activities [5,7] and has been found to increase significantly following spinal cord injury [6]. The role of PLA_2 _has also been documented in schizophrenia, brain trauma and Alzheimer's disease [8,9] besides global and focal ischemia in animal models [10]. Hippocampal slices subjected to oxygen and glucose deprivation (OGD) have been found to show an increase in PLA_2 _activity and a concomitant death of neuronal cells. Inhibition of cPLA_2 _has been found to result in an enhanced survival of the hippocampal neurons [11]. Evidently, cPLA_2 _knock-out mice subjected to focal cerebral ischemia showed significant reduction in infarct volume and the extent of neurological impairment [12]. Furthermore, Strokin et al [13] demonstrated that inhibition of iPLA_2 _during OGD could render neuroprotection to the hippocampal slice cultures. Furthermore, simultaneous inhibition of cPLA_2 _and sPLA_2 _activities have also been shown to improve survival of glial cells subjected to ischemic injury [14].

The snake venom PLA_2 _belongs to the Ca^2+^-dependant secretory PLA_2_. Venom phospholipases A_2 _possess an enzymic activity and a wide variety of (patho)pharmacological activities such as antiplatelet, anticoagulant, hemolytic, neurotoxic (presynaptic), myotoxic, edema-inducing, hemorrhagic, cytolytic, cardiotoxic as well as an ability to bind antagonistically to muscarinic acetylcholine receptor (mAChR) [15-17]. The snake venom phospholipases are divided into two main groups, group I and group II, based on their primary structures [1]. The group I PLA_2 _is found in abundance in the venom of cobras, kraits and sea snakes, while group II PLA_2 _is common in vipers and pit vipers. The cobra venom PLA_2 _belongs to group IA that is similar to the pancreatic type group IB protein but without the signature pancreatic loop structure. Venom of *Naja sputatrtix*, a Malayan spitting cobra, comprises of three isoforms (2 neutral and 1 acidic) of group 1A PLA_2_. One of the neutral forms, nPLA-1 (nPLA) is a highly potent anticoagulant protein that exhibits relatively high enzymic activity [18-20]. This protein has also been shown to possess an ability to bind to all muscarinic receptor subtypes (m1-5) with a higher affinity to the m5 subtype [16].

In this report, in contrast to the reported detrimental effects of mammalian phospholipase A_2 _to the central nervous system, we demonstrate that neutral PLA_2 _(nPLA) from *Naja sputatrix *could reduce neuronal cell death and afford neuroprotection to rat brain subjected to transient focal ischemia. Furthermore, OGD induced tissue injury has also been found to be reduced in the presence of nPLA. Real-time quantitative gene expression analysis showed that the pro-survival and anti-apoptotic genes have been upregulated. Both caspase 3 (*in vitro*, cell culture) and TUNEL (*in vivo*, brain slices) assays showed that apoptotic cell death can be reduced upon treatment with nPLA.

## Methods

### Purification of nPLA

Phospholipase A_2 _(nPLA) was purified from *Naja sputatrix *crude venom (Sigma Chemicals Co., USA) using Sephadex G-100 gel filtration followed by reverse phase high performance liquid chromatography (RP-HPLC). The protein fractions were characterized as described by Armugam et al [18] and quantitated using the Bradford assay (Bio-Rad Laboratories, CA).

### Transient focal cerebral ischemia

Male Sprague-Dawley rats (200-300 g) were obtained from the Laboratory Animal Centre (National University of Singapore, Singapore) and maintained on an *ad libitum *intake of standard laboratory chow and drinking water. All animals were handled according to the guidelines given by the Council for International Organization of Medical Sciences (CIOMS) on animal experimentation (WHO, Geneva, Switzerland) and the National University of Singapore (IACUC/NUS) guidelines for handling laboratory animals. Animals were anaesthesized and left middle cerebral artery occlusion (MCAo) was performed as described by Longa et al [21]. The occlusion was confirmed with the real-time measurement of cerebral blood flow (CBF) in the territory of the middle cerebral artery (MCA) using the Laser-Doppler flowmetry (OxyFlo, Oxford Optronix) and the signals were digitized using a 4-channel Powerlab 4SP (ML760) and recordings were displayed with Chart 5 software (ADInstruments Pty Ltd, Australia). Reperfusion was initiated by suture withdrawal after 60 min. nPLA (0.075-0.25 μg/g body weight) was injected intravenously via the femoral vein at various times of post-occlusion.

Recombinant tissue plasminogen activator (tPA; Boehringer Ingelheim, Germany) was infused intravenously at a concentration of 10 μg/g body weight over a period of 60 min, starting 30 min post-occlusion. MK801 (Sigma-Aldrich Co., USA) was administered intraperitoneally in 3 doses: 2.5 μg/g body 30 min prior to MCAo followed by 1.25 μg/g body weight at 6 and 14 hr post-occlusion. Corresponding controls were treated with sterile saline and all animals were sacrificed after a total of 24 hr reperfusion period.

Whole brains were rapidly removed and snap-frozen in liquid nitrogen. Frozen brains were stored at -80°C until use. Consequently, there were 5 treatment groups: [1] Sham-operated (sham-op, n = 7); [2] Transient MCAo (MCAo) for 60 min and administered with 100 μl saline (MCAo, n = 10); [3] Transient MCAo for 60 min and administered with nPLA intravenously (MCAo+nPLA, n = 20); [4] Transient MCAo for 60 min and administered with tPA intravenously (n = 6); [5] Transient MCAo for 60 min and administered with MK801 intraperitoneally (n = 6).

### Quantitation of the ischemic infarct volume

Whole rat brains were sliced coronally into 1 mm slices and incubated in 2, 3, 5-triphenyltetrazolium chloride (TTC; Sigma, St Louis, MO, USA) and fixed in 10% buffered formalin. Stained brain slices were scanned and the image was analysed using Scion Image analysis software for measurement of the infarct volume. The infarct size was determined according to Engelhorn et al [22]. Histopathological changes in the brains were evaluated from paraffin embedded brain slices. Cell morphology assessment was carried out using the haematoxylin and eosin staining. The degree of apoptotic neuronal cell death was assessed by TUNEL staining using the ApopTag^® ^Peroxidase *In situ *Apoptosis Detection kit (Chemicon International, USA) according to manufacturer's protocol.

### RNA isolation and real-time quantitative PCR

Total RNA was isolated from brain tissues (frozen or fresh) by a single-step method using TRIzol^® ^Reagent (Invitrogen, USA). The RNA samples were treated with RNase-free DNase at 37°C for 20 min and stored at -80°C until further use. Reverse transcription of total RNA and real-time PCR studies using SYBR-Green chemistry (Applied Biosystems, USA) were carried out according to Cher et al [23]. A dissociation protocol was carried out at the end of each experiment that was performed in triplicates and repeated at least 3 times for each case.

### Organotypic hippocampal slice culture

Organotypic hippocampal slice cultures were prepared as described previously [24] with slight modifications. Briefly, hippocampii from 6 to 8 days old Sprague Dawley rat pups were dissected and sliced to 350 μm thickness and placed in ice-cold growth medium (50% MEM, 25% HBSS, 25% heat inactivated horse serum, 5 mg/mL glucose, 1 mM glutamine, 1.5% fungizone). The sliced tissues were placed onto semiporous membranes (culture plate inserts; Millicell, Milipore Co, Bedford, MA) and grown for 10 to 14 days at 37°C with 5% CO_2 _enriched atmosphere before subjecting to oxygen and glucose deprivation (OGD) studies. Propidium iodide (PI; 5 μg/ml) was included in the culture media during the experiments to trace the damage on the tissues.

### Oxygen-glucose deprivation (OGD)-mediated ischemic injury

Organotypic hippocampal cultures were grown in basal medium (75% MEM, 25% HBSS, 1 mM glutamine, and 1.0% penicillin-streptomycin) and transferred to six-well plates containing glucose-free medium (75% glucose-free MEM, 25% HBSS, 1 mM glutamine and 1.0% penicillin-streptomycin) saturated with 95% N_2_, 5%CO_2 _and placed into an anaerobic chamber at 37°C and 100% humidity for 90 mins. nPLA (0.0375 μM, 0.075 μM and 0.15 μM), was dissolved in distilled water and added to the medium immediately before exposure to OGD. After 90 mins, the cultures (culture plate semiporous inserts) were then transferred to a fresh 6 well plate containing pre-warmed serum free medium with 5 μg/mL propidium iodide [24] and incubated in the CO_2 _chamber for 24 hours. The hippocampal slices were viewed under confocal microscope and analysis of damaged CA1 hippocampal neurons following OGD was carried out using ImagePro Plus Software (Media Cybernetics, Silver Spring, MD, U.S.A.). Hippocampal culture in serum free media was used as controls in all experiments.

### Astrocytoma (CRL1718™) cell culture

The human astrocytoma cells (CRL-1718™, ATCC) were cultured in RPMI 1640 media (Hyclone Laboratories, USA) supplemented with 10% fetal bovine serum (FBS), 1% L-glutamine and 1% Penicillin-streptomycin (GIBCO-BRI, Gaithersburg MD, USA) and maintained in a 37°C incubator with 5% CO_2_. Cells were checked regularly under the light microscope and were divided into appropriate culture plates when they reached 70-80% confluence. Cells were subcultured at a density of 1.0 × 10^5 ^cells (24-well plates) or 1.25 × 10^4 ^cells (96-well plates) as required and subjected to either staurosporine or OGD treatment(s).

### Detection of TNF-α, cytotoxicity assay and caspase assay

Detection of TNF-α (inflammation marker) in the blood serum of rats subjected to MCAo (treated with either nPLA or LPS) was carried out using the Chemikine™ Rat TNFα Sandwich ELISA kit (Chemicon International, USA) according to manufacturer's protocol. Blood samples from Sprague Dawley rats (n = 6) with and without MCAo were administered intravenously with saline (100 μl) or nPLA (0.15 μg/g body weight) or intraperitoneally with bacterial lipopolysaccharide (LPS; 2 μg/g body weight). LPS was used as a positive control to induce inflammation and for the release of TNF-α.

In organotypic hippocampal slice culture studies, cell death measurement was carried out using the lactate dehydrogenase (LDH) assay (Roche Life Science, Germany). Briefly, the LDH activity in the culture media was measured spectrophotometrically at 490 nm using a multi-plate scanning spectrophotometer (Model 680 Microplate Reader, Bio-Rad Laboratories, CA) at an end point of 30 min. Caspase-3-like protease activity was measured by fluorometric assay (Axxora Life Sciences Inc., USA). DEVD-AFC was used as substrate for caspase activity of the cell lysate and the cleaved AFC (7-amino-4-trifluoro-methyl coumarin) was measured in a spectrofluorometer at 400/505 nm (excitation/emission).

### Microarray analysis

Total RNA isolated from sham, MCAo and MCAo+nPLA brains was pooled to minimize inter-individual variation and hybridized to each array of the RAE-230A or U34A GeneChip™ according to protocols described in the GeneChip™ expression analysis package (Affymetrix, CA). Each chip represented ~15,900 genes and ESTs and data for each treatment were scaled to an average intensity of 800. Probe sets designated 'absent' in all treatments by the analysis software were discarded and only genes whose expressions were changed by 0.6-fold or greater in each pairwise comparison (between sham-op and MCAo or between MCAo and MCAo+nPLA) were deemed significant. Differentially expressed genes (p < 0.025) were classified according to their biological functions as described in the NetAffx Analysis Center  and PubMed  databases. The data discussed in this publication have been deposited in NCBI's Gene Expression Omnibus [25] and are accessible through GEO Series accession number GSE17929, .

### Statistical analysis

All statistical analysis was carried out using single-factor ANOVA followed by (a) Dunnett multiple comparison tests for MCAo brain slice analysis and (b) for all other *in vitro *experiments, pairwise comparisons were carried out using unpaired Student's *t*-test.

## Results

### nPLA reduces infarct volume in MCAo rat model

The nPLA native protein, was purified from *Naja sputatrix *according to the methods described by Armugam et al [18]. The purity and identity of this protein was confirmed by mass spectrometry and N-terminal sequencing before using in this study. *Naja sputatrix *venom contains three (2 neutral and 1 acidic) isoforms of PLA_2_. Their molecular mass is approximately 14 kDa and all of them have anticoagulant and phospholipase activity [20]. Among them, the nPLA is a most potent anticoagulant, with an enzymic activity and also competes with atropine for the muscarinic receptor [16].

**Figure 1:**
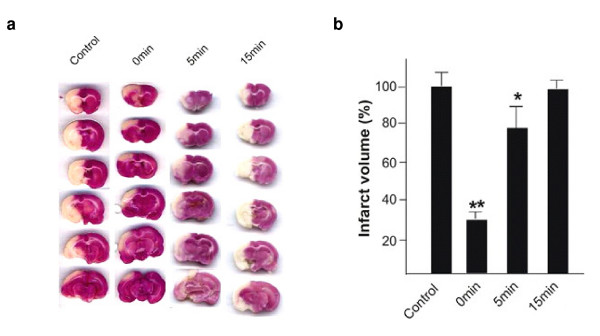
**Histological analysis of brain sections**. (**a**) TTC stained coronal brain sections (2 mm thick) of rats treated with 0.15 μg/g body weight nPLA intravenously at 0 min, 5 min and 15 min post-occlusion (n = 6). Surviving cells stained red whilst dead cells remained white. (**b**) Infarct volumes are expressed as a percentage of the vehicle control ± SEM. *, *p *< 0.05; **, *p *< 0.01 using single-factor ANOVA followed by Dunnett multiple comparison test.

The anti-ischemic and neuroprotective property of the nPLA was initially observed *in vivo*, in rat model subjected to transient middle cerebral artery occlusion (MCAo). nPLA was administered intravenously, at doses of 0.075 μg/g, 0.15 μg/g, 0.25 μg/g, body weight (0.15 μg/g = 0.25 LD_50 _= 1 μg/ml = 0.075 μM) immediately 0 min, 5 min and 15 min after MCAo. However, the highest neuroprotective effect (measured initially as reduction in the infarct volume) has only been observed at the 0.15 μg/g rats and therefore all experiments were conducted at this concentration of nPLA_2 _(Figure 1a &1b). The administration of nPLA immediately upon occlusion (0 min) resulted in a significant reduction (~70% reduction) in infarct size, as observed by triphenyltetrazolium chloride (TTC) staining of serial brain sections (Figure 1a). Ischemic damage was markedly attenuated in the striatum and cortex of the brain, with more pronounced protection in striatal tissue. Administration of nPLA at 5 min post-MCAo also reduced the infarct size but to lesser extent (~15% reduction; Figure 1b). No protection was observed at 15 min or later. The abilities of tPA and a non-competitive antagonist of the NMDA receptor, Dizocilpine (MK801) to reduce infarct volume was used as controls and were compared to that of nPLA. Intravenous infusion of 10 μg/g body weight tPA did not significantly reduce infarct volume after transient ischemia (see Additional file 1a&1b). The inability of tPA to prevent neuronal death is perhaps not surprising since its efficacy has mainly been demonstrated in embolic stroke models [26] or after mild transient focal ischemia [27]. On the other hand, the intra-peritonial administration of MK801 in three doses (total of 5.0 μg/g), at 2.5 μg/g body weight about 30 min prior to MCAo and followed by 1.25 μg/g body weight at 6- and 14 hr post-occlusion [28] significantly reduced infarct volume to 61.1% ± 8.6% as compared to the vehicle control (see Additional file 1a&1b). The extent of neuroprotection conferred by MK801 is however lower than that observed for nPLA (33.2% ± 5.1%) administered immediately post-occlusion. Nevertheless, the MK801 conferred neuroprotection was significantly higher than that conferred by nPLA given after 5 min of MCAo (78.3% ± 10.8%; Figure 1b & see Additional file 1b). It should be noted that MK801 was administered at 3 time points (2.5 μg/g and twice at 1.5 μg/g) while the nPLA was administered only once at 0.15 μg/g. Thus the efficacy of nPLA mediated protection can be considered to be more pronounced than that of the positive control, MK801. TNF-α has been demonstrated to be elevated upon MCAo and this increase in TNF-α activity results in increase of the endogenous sPLA_2 _expression/activity that is implicated in neuronal damage in ischemic models [29]. Intraperitoneal administration of bacterial LPS (positive control for TNF-α induction) dramatically increased plasma TNF-α levels (232 ± 25.1 pg/ml) as compared to the saline-treated control (2.6 ± 0.05 pg/ml). In contrast, nPLA_2 _failed to induce significant TNF-α release or expression in both normal and ischemic rats (4.1 ± 0.03 pg/ml and 1.0 ± 0.01 pg/ml respectively; see Additional file 1c). Thus, the 0.25 LD_50 _nPLA (0.15 μg/g body weight in rats) does not induce inflammation or cytotoxicity as previously observed by Tan and Armugam [20].

### Reduction in apoptotic cell death upon nPLA treatment

**Figure 2:**
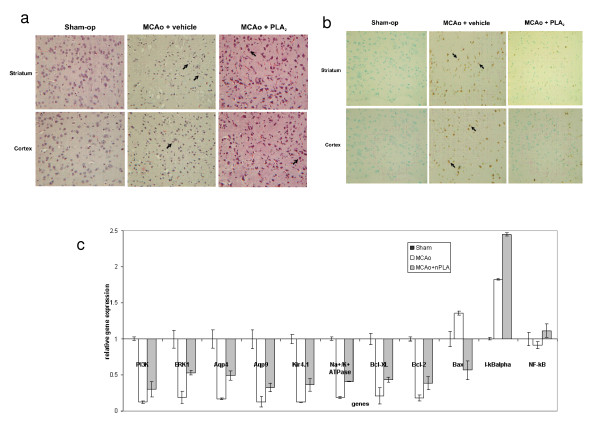
**Anti-apoptotic effect of nPLA (a) Histological and TUNEL analysis of brain sections**. High power (×120) photomicrographs of haematoxylin and eosin-stained **(b) TUNEL-stained striatal and cortical tissue**. Nuclei are indicated by arrows. Rats subjected to transient MCAo were injected intravenously with either vehicle or 0.15 μg/g body weight nPLA. Sham-operated animals underwent the entire surgical procedure other than suture insertion. **(c) Quantitative gene expression (real-time PCR) analysis**. Gene expression analysis on selected genes for sham operated (Sham), MCAo and MCAo+nPLA brain samples. All measurements were performed in triplicate. Values are expressed as fold change ± SEM, * *p *< 0.05; ** *p *< 0.01 by unpaired Student's *t*-test.

**Figure 3:**
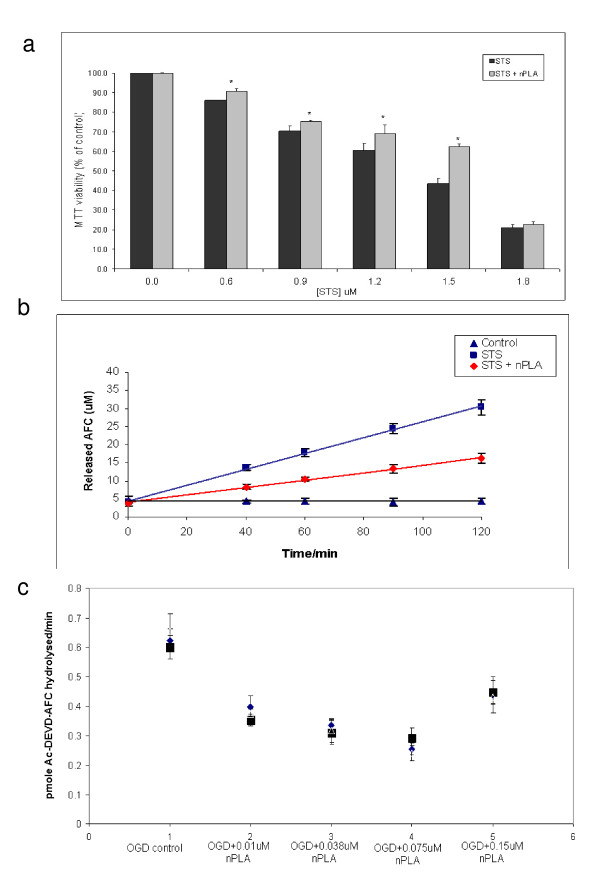
**(a) Effect of nPLA (0.01 μM) on dose-dependent STS-induced cell death in astrocytoma cells (CRL1718™)**. Cell viability assay was carried out following 16 hrs exposure to various concentrations of STS or STS + 0.01 μM nPLA. Asterisks denote difference from its each individual STS concentration treatment. Values are expressed as percentage of viability (to the respective treatment without nPLA) ± SEM, * *p *< 0.05 by unpaired Student's *t*-test (n = 9). **(b) Effect of nPLA on STS-induced cell death by caspase-3 activity assay**. Control: no reagents, STS: 1.5 μM STS, STS + nPLA: 1.5 μM STS+0.01 μM nPLA. Data shown are mean ± SEM (n = 18). **(c) Effect of nPLA on CRL1718™ astrocytoma cells subjected to OGD**. Cells were subjected to 2 hr OGD and nPLA (0.038 μM or 0.01 μM) was added in the culture during the ischemic period. Cell viability assay was carried out following a 24 hr of reperfusion. Caspase-3 assay was carried out on the respective cell lysates. Data shown are mean ± SEM (n = 3, conducted in triplicates, *p *< 0.05, two tail Student's *t *test).

The paraffin-embedded rat brain (MCAo, MCAo+nPLA) sections were stained with haematoxylin and eosin and cellular morphology was evaluated using light microscopy. Extensive tissue damage and edema were observed in the striatum and cortex of MCAo brain slices (Figure 2a). Neurons appeared shrunken and nuclei were dysmorphic and pyknotic, consistent with apoptotic cell death [30]. TUNEL assay was carried out to assess the extent of apoptosis in ischemic brains (Figure 2b). Control cells appeared green whereas almost all the neurons in the striatum and cortex of ischemic brains (MCAo) exhibited apoptosis and their nuclei were stained brown. The degree of apoptosis was greatly reduced by the administration of nPLA immediately post-occlusion as evidenced by the substantial reduction in number of apoptotic cells. Furthermore, real-time PCR analysis on selected genes encoding apoptosis related proteins (Bcl-2, Bcl-_XL _and Bax) and the survival pathway mediating proteins (NF-κB and IκB) as well as the signalling pathway proteins (PI3K, Erk1), and ion and water channel proteins (Kir_4.1_, Na^+^K^+^-ATPase, Aqp4 and Aqp9), showed (Figure 2c) that the nPLA treatment reduces the effects of MCAo-mediated neuronal/cell damage via promoting cell survival/protective mechanisms. Similarly, we have also observed that staurosporine (STS)-mediated cell death in human astrocytoma cells (CRL1718™) could be alleviated by 0.01 μM nPLA (Figure 3a). The reduction in cell death has also been found to be accompanied by a decrease in caspase-3 activity (Figure 3b). Furthermore, inclusion of 0.038 μM nPLA in the basal media during OGD, protected the astrocytoma cells from cell death. Improved cell viability has always been accompanied by a significant reduction in caspase-3 activity (Figure 3c).

### nPLA-mediated neuroprotection in *in vitro *studies

**Figure 4:**
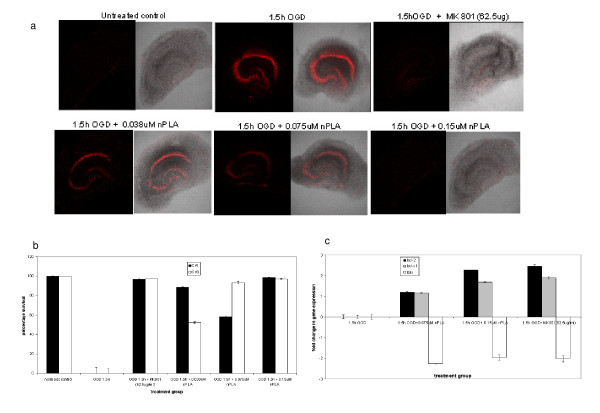
**Organotypic hippocampal culture subjected to OGD**. **(a) **Slices subjected to oxygen-glucose deprived (OGD) condition in the presence of nPLA (0.037 μM-0.15 μM) and propidium iodide (5 μg/ml). Confocal microscopy images show that brighter areas correspond to a high propidium iodide [24] uptake level indicating a higher density of dead or damaged cells. **(b) Percentage of cell survival, **calculated based on PI intensity on each CA1 and CA3 regions. Each point represents the mean ± SEM (n = 8). #; *p *< 0.05 and *; *p *< 0.01. **(c) Quantitation of gene expression using real-time PCR**. Statistical analysis was performed using one-way analyses of variance (ANOVA; *; *p *≤ 0.005.) and a t-test to determine significance between groups for LDH assay (n = 6) and real-time PCR (n = 9).

nPLA-mediated neuroprotection was also observed in the organotypic hippocampal culture subjected to OGD (Figure 4a). Almost complete (97 ± 0.81%) protection was seen at 0.15 μM nPLA, which is similar to the protection induced by positive control, MK801 (Figure 4b). LDH assay showed that nPLA could reduce the cytotoxicity induced by OGD. The Bcl-2 (1.2 ± .018 and 2.27 ± 0.02 fold) and Bcl-_XL _(1.17 ± 0.025 and 1.69 ± 0.03 fold) genes were upregulated whereas Bax (-2.27 ± 0.016 and -1.97 ± 0.129 fold) was found to be downregulated in the organotypic hippocampal slice culture in OGD conditions at both 0.075 μM and 0.15 μM nPLA respectively (Figure 4c). Similarly, concurrent (during glutamate incubation) and post-treatment with nPLA showed reduced neuronal injury in a dose-dependent manner in hippocampal slice culture (both the CA1 and CA3 regions) subjected to glutamate induced excitotoxicity (data not shown).

### Global gene expression analysis

Global gene expression analysis by oligonucleotide microarray in MCAo and MCAo+nPLA showed a total of 1455 genes (filtering criteria; 0.6<SLR<-0.6 and detection p value < 0.025).

**Figure 5:**
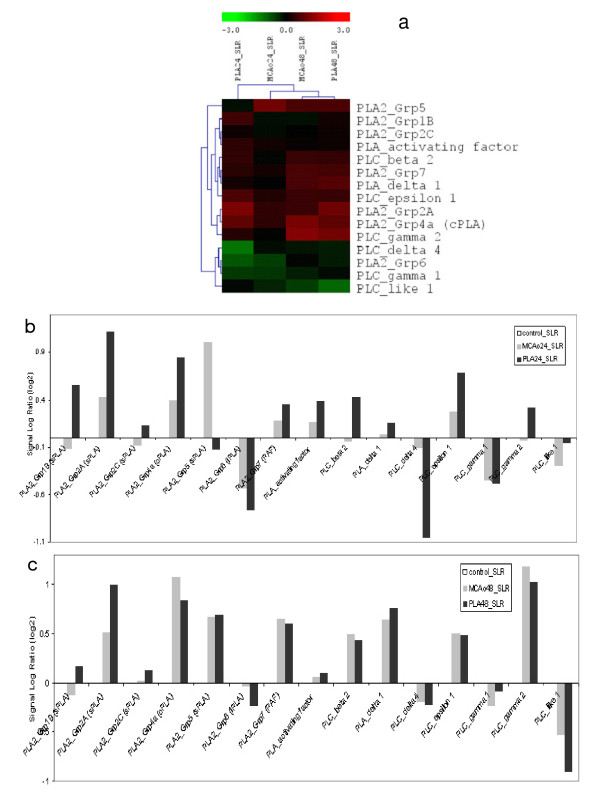
**Microarray analysis on the endogenous phospholipases**. **(a) Hierachical clustering **of all the phospholipases detected in our global gene expression studies. Signal log(2) ratio (*vs *normal control) of the average signal intensities (detection p value < 0.01) were used to construct the dendrogram and the genes and samples were clustered based on Euclidean distance and average linkage method (TIGR MultiExperiment Viewer; [52]). **(b) **expression of endogenous phospholipases at 24 hour reperfusion **(c) **expression of endogenous phospholipases at 48 hour reperfusion. **Control**, sham operated rat brain; **MCAo**, rats subjected to middle cerebral artery occlusion and reperfused for either 24 hr or 48 hr; **PLA**, MCAo rat administered with nPLA (0.15 μg/g rats) and allowed reperfusion for 24 hr or 48 hr.

Among these, the endogenous phospholipases (PLA_2 _and PLC) have been found to be upregulated upon treatment of the the MCAo rats with nPLA (Figure 5a&5b). The expression of cPLA_2 _(PLA_2_-4A), reported to contribute to the pathophysiological conditions and neurological deficits during stroke, has also been found to be significantly higher in the nPLA treated rats. On the other hand, the iPLA_2 _(a calcium-independent Group 6 cytosolic PLA_2_), has been found to be downregulated in both the control and nPLA treated MCAo rats. It is noteworthy that the PLCs (with exception to PLCδ4, PLCγ1) that are involved in Ca^2+ ^dependent signal transduction activities remained highly upregulated in the nPLA treated animals. However, following a prolonged (48 hr) reperfusion the expression of all the endogenous phospholipases except PLA_2_-1B, PLA_2_-2A and PLA_2_-2C reached a basal level comparable to that of the control. nPLA treatment showed an upregulation of PLA_2_-1B, -2A and -2C and a downregulation of endogenous iPLA_2_(Figure 5c).

The microarray dataset when clustered by k-means clustering using the Genesis software package [31] gave 15 clusters of which clusters 1 and 6 (see Additional file 2) represented the genes that were fully restored to sham levels following nPLA treatment (see Additional file 3 &4).

The genes in cluster 1 (downregulated) and cluster 6 (upregulated) during MCAo revert to normal (Sham) level of expression upon nPLA treatment. The gene ontology classes can also be seen to be quite similar in both clusters 1 & 6. The major sites (cellular component) of action of nPLA appear to be the membrane and nucleus, implicating involvement of receptors and signaling pathways. This is further observed in pathway analysis using Gene Ontology (GO) and GENMAPP program [32] where most of the pathways involved are signalling pathways. Cluster 6 contains genes that are localized in the ribosome as well.

Nucleotide binding (ATP binding) is the most common functional GO class found for both clusters. Interestingly there are several ion binding related GO classes such as calcium and zinc ion binding that are common to both clusters, implicating the involvement of nPLA in the regulation of ionic levels of the cells. G-protein coupled receptor (GPCR) protein signalling pathway and intracellular protein transport are among the several common processes that were observed in both the clusters 1 & 6.

The pathways involved in apoptosis (Epithelial growth factor receptor (EGFR) 1, TNF-alpha-NF-kB and MAPK signalling pathways) and inflammation (Interleukin and T-cell receptor and B-cell receptor signalling pathways), were observed to be highly affected by nPLA treatment in MCAo rats. NFκB1 which is upregulated in nPLA treatment directly inhibits apoptosis (see Additional file 5). EGFR1 pathway regulates apoptosis and many genes of this pathway can be seen in clusters 1 and 6 (see Additional file 6). Hspa1a also known as HSP70 that inhibits apoptosis was found to be further upregulated upon nPLA treatment than MCAo (see Additional file 7). NFkb1 which is upregulated only in nPLA treatment is known to enhance cell survival. Thus, nPLA treatment appeared to protect the cell by anti-apoptotic as well as cell survival mechanisms.

## Discussion

### nPLA reduces infarct volume and protects neurons from cell death

In this report, we demonstrate that nPLA could reduce apoptotic cell death and render neuroprotection against cerebral ischemia. Intravenous administration of nPLA to the MCAo rats at 0 min and 5 min post-occlusion reduced infarct volumes to 33.2% and 78.3% respectively as compared to the vehicle control. Saluja et al [33] observed significant increase in cPLA_2 _activity and expression after 10 mins and 20 mins upon onset of global ischemia. Furthermore, in an *in vitro *study, cPLA_2 _activity was not detected in the absence of calcium ion [34]. Thus, these reports could explain our observation that administration of nPLA at only 0 min (initiation of occlusion) and 5 mins and not after 15 mins of occlusion, showed neuroprotection in our rat MCAo model. Significant reduction in ischemic damage has also been observed in histological analysis of the brain slices, where many cell nuclei exhibited normal morphology in nPLA treated MCAo rat brain. Neurons within the ischemic core die largely by means of a necrotic mechanism as a result of the excitotoxicity cascade triggered by energy depletion while damage within the penumbra is mediated by mechanisms such as apoptosis. During cerebral ischemia, sub-lethal injury to neurons favours the initiation of apoptosis in the penumbral neurons [35]. The nPLA-mediated protection has been found to target the area/region where cells are undergoing apoptosis and consequently reducing cell death as observed in the TUNEL assay. Daniel and DeCoster [36] have demonstrated that TUNEL staining could be used as apoptosis marker and is significant at later times (>4 hr and <26 hr) of cell death. Hence, nPLA appears to possess the ability to protect, possibly the penumbra region from ischemic damage. Furthermore, nPLA-mediated 'protection' was also observed in human astrocytoma cells (CRL-1718™) exposed to staurosporine (STS) where increased cell viability and reduction in caspase 3 activity was observed upon nPLA treatment (Figure 3a &3b). Furthermore, significant reduction in FITC-Annexin V stained cells were observed in the astrocytoma exposed to STS and treated with 0.01 μM nPLA. Hoechst33342 staining showed that lesser number of DNA fragmentation occurs in the presence of nPLA. Thus indicating that nPLA could be mediating cell protection by reducing the apoptotic cell death.

Hippocampal slice cultures subjected to OGD also showed that 0.037 μM nPLA promoted about 60% and 95% survival at the CA1 and CA3 regions respectively, whereas 0.075 μM nPLA showed 95% protection for both regions. The protection mediated by nPLA (0.075 μM) was similar to that observed for MK801, a selective non-competitive antagonist of NMDA receptor. MK801 was used as a positive control for neuroprotection as it has been shown to be highly neuroprotective in both models of ischemia and hypoxia [37,38]. We have also observed that the protection shown in the presence of nPLA is solely mediated by nPLA and not by intracellular PLA_2 _(iPLA_2_, cPLA_2 _or sPLA_2_). This is also because (a) similar protection could not be observed in the vehicle treated control slices subjected to OGD and (b) the endogenous PLA_2_s are known to mediate cell death rather than protection in organotypic hippocampal slice cultures subjected to OGD [11,13,14].

Gilroy et al [39] have also demonstrated that iPLA_2 _is highly expressed at the onset phase of an acute inflammation with comparatively lower levels of sPLA_2 _(Groups 2A and 5) as well as cPLA_2 _(Group 4) while the sPLA_2 _and cPLA_2 _were the predominant isoforms expressed during lesion resolution. Inhibition of both the cPLA and iPLA has been proposed to be beneficial in reducing infarct volume and increasing the neurological activities of mice subjected to MCAo [10,12] as well as increasing survival and neuroprotection in *in vitro *experiments [11,13]. The global gene expression data show that nPLA administration during MCAo reduces the iPLA_2 _but increases the cPLA_2 _and sPLA_2 _expression (1B, 2A, 2B). In contrast to the general observation that endogenous PLA_2 _promotes pathophysiological condition, Forlenza et al [40] reported that reduced endogenous PLA_2 _(cPLA and iPLA) activity could impair neuronal viability and the functional integrity of both calcium-dependent and calcium-independent cytosolic PLA_2_. Endogenous sPLA_2_-X and human sPLA_2_-III have been reported to promote neurite outgrowth in PC12 cells [41,42], an effect that is not observed with the administration of sPLA-1B or sPLA-IIA. It is noteworthy that the nPLA belongs to the group 1A (sPLA-IA) which is similar to the sPLA-1B but without the signature pancreatic loop.

We have also shown that nPLA could protect cell death induced by glutamate (see Additional file 8) in the hippocampal slice culture as well as in the astrocytoma cell culture (CRL-1718™; Figure 2d-f). Furthermore, in a separate *in vitro *study on astrocytes and hippocampii using glutamate receptor agonists and antagonists as well as an inhibitor to phospholipase activity (4-bromophenacyl bromide), we have found that the nPLA mediated neuroprotection is exerted via the mGluR, specifically mGluR1 and not by its phospholipase activity (unpublished data).

Quantitative gene expression analysis on the MCAo (+nPLA) ipsilateral brain (Figure 2c) and hippocampal tissues subjected to OGD (+nPLA) (Figure 4c) showed that the pro-survival (NFκB) and anti-apoptotic (Bcl-2 and Bcl-_XL_) genes were up-regulated while the pro-apoptotic Bax gene was down-regulated upon nPLA administration in both the *in vivo *and *in vitro *studies. Bax homodimer has been reported to activate apoptosis while the heterodimer (with Bcl-2 or Bcl-_XL_) is known to inhibit the process [43]. Elevated intracellular ratio of Bax to Bcl-2 occurs during increased apoptotic cell death [44]. Similarly, over-expression of Bcl-2 in *in vivo *ischemic studies resulted in reduced apoptotic cell death [45,46]. Hence, the quantitative real-time PCR results on the brain sample subjected to MCAo and hippocampal slice culture subjected to OGD, further support that apoptotic cell death is reduced upon treatment with nPLA. The high expression of both anti-apoptotic genes (Bcl-2 and Bcl-_XL_), could possibly result in Bax/Bcl-2 or Bcl-_XL _heterodimerization, thereby inhibiting apoptosis and promoting neuroprotection. Similarly, Neuroprotectin D1, derivative of docosahexaenoic acid (DHA), that promotes strong neuroprotection and neurotrophic activity following ischemia and reperfusion, also up-regulates Bcl-2 and Bcl-xL. Neuroprotectin D1 was also observed to inhibit the caspase-3 activation [47]. However, nPLA improved cell viability and survival in astrocytoma cells subjected to OGD. The increase in cell viability was accompanied by significant reduction in caspase-3 activity.

Consistently, reduction in caspase activity and increased in cell viability have also been observed in staurosporine-mediated apoptosis in astrocytoma cells treated with nPLA (Figure 3a&3b). Oligonucleotide/DNA microarray analysis also suggests that nPLA treatment in MCAo rats reduce the impact of MCAo-mediated cellular damage to normal (sham) level via inhibiting or reducing the effect of apoptosis and inflammatory mechanisms, thus supporting an anti-apoptotic regulation as a possible mechanism of action for nPLA-mediated neuroprotection, which is also consistent with our TUNNEL assays and Real-time PCR analysis.

### Regulation of water and ion channel genes

Apoptotic volume decrease (AVD), the earliest morphological event of apoptosis that is depicted by pronounced cell shrinkage is believed to involve regulation of water and ion channels. During AVD, intracellular ion concentrations are altered following inhibition of Na^+^/K^+^ATPase in conjunction with a transient Na^+ ^accumulation followed by the extrusion of both Na^+ ^and K^+ ^ions from the cell. Decreased intracellular K^+ ^is in turn required for the activation of the apoptotic/caspase cascade and optimal nuclease activity [48]. Water movement during the AVD is mediated primarily via aquaporins and that plasma membrane water permeability directly affects the rate of apoptotic progression [49]. Aquaporins have been shown to play a pivotal role in the formation and clearance of fluid (brain edema) during cerebral ischemia. The Aqp4 is a water specific mercury insensitive water channel that is found abundantly in brain and the Aqp9 is an aquaglyceroporin that conducts urea, lactate, arsenite, purine and pyrimidines, besides water molecule [50]. Aquaporin 4 and 9 have been shown to be down-regulated in experimental ischemic rat brain, while the Aqp4 knockout mice subjected to MCAo showed smaller infarct volume. The regression of ischemic infract also required an up-regulation of Aqps [51]. Interestingly, in our study, both these aquaporins (Aqp4 and 9) were up-regulated upon nPLA administration. Up-regulation of Aqp9 therefore suggests that the lactate and other solutes that were formed during the ischemic injury may be channelled out of the cell via Aqp9, thus could prove beneficial in neuroprotection. Notably, the Kir_4.1 _and Na^+^/K^+^ATPase genes were also upregulated upon nPLA administration in the MCAo rat. Similarly, we have also observed that expression of the aquaporin genes as well as the K_ir_4.1 and Na^+^/K^+^ATPase in astrocytoma cells subjected to OGD (2 hr) were reversed in the presence of nPLA. Expression of genes involved in cell survival promoting pathway (PI3K, ERK1 and NFkB) have also been significantly upregulated with nPLA administration, indicating that an anti-apoptotic, homeostatic (ionic/physiologic) and cell survival regulation are being triggered in nPLA-mediated neuroprotection.

## Conclusion

Snake venom PLA_2 _is known for its pathopharmacological activities. We have shown here that nPLA, a potent toxin isolated from *Naja sputatrix *venom, could reduce neuronal cell death and promote cell survival both under in vivo and in vitro ischemic conditions. Its beneficial effects could be seen at a sublethal in vivo dose of 0.15 μg/g rats (0.25 LD_50_) and at a concentration of 0.15 μM under in vitro conditions.

## Authors' contributions

AA purified the venom PLA_2 _and characterised the protein and carried out the molecular genetic studies. CDNC & LKY carried out the animal experiments (MCAo rat model) and (statistical) analysis. DCIK carried out the in vitro experiments using the hippocampal tissues and astrocytes respectively, the gene quantitation study (real-time PCR) and analysed the results. DH provided training on the creation MCAo rat models and assisted in the initial phases of the experiments. KJ conceived the study plans, coordinated the project and assisted in the preparation of the manuscript. All authors have read and approved the final manuscript.

## Supplementary Material

Additional file 1 **Rats subjected to MCAo and treated with tPA and MK801**. **(a) **Infarct volumes were expressed as a percentage of the vehicle control ± SEM. **, p < 0.01 by unpaired Student's t-test. **(b) **TTC stained coronal brain sections (2 mm thick) from rats treated with tPA and MK801 (n = 6). **(c) **Serum TNF-α level. Rats were either treated intravenously prior to or after transient MCAo (n = 6 per treatment group). Results are the mean of duplicate experiments and expressed as concentration of serum TNF-α ± SEM. **, p < 0.01 by unpaired Student's t-test. Click here for file

Additional file 2 **Oligonucleotide microarray data analysis**. The microarray dataset was clustered by K-means clustering using Genesis Software. The first data point denotes MCAo treatment and the second data point denotes MCAo+PLA treatment. Of the total 15 clusters obtained only Clusters 1 and 6 were selected. Click here for file

Additional file 3 **NetAffex & GenMAPP analysis of microarray data**. Total of 1455 genes were selected from the oligonucleotide microarray raw data based on our filtering criteria (0.6<fold change<-0.6 and detection p < 0.025). This gene list was further subjected to NetAffex analysis, K means-clustering, GenMAPP and Gene Ontology analyses. The microarray dataset was clustered by K-means clustering. Total of 15 clusters were obtained. Clusters 1 and 6 showed that the genes affected by MCAo were normalised to sham levels upon nPLA administration. Click here for file

Additional file 4 **Gene Ontology analysis of genes in Cluster 1 & 6**. The gene list of the clusters 1&6 were used to perform a Gene Ontology and pathway analysis using GENMAPP based on the number of genes involved in each GO term/pathway. Click here for file

Additional file 5 **Inhibition of apoptosis pathway by NFκB1**. NFκB1 gene is upregulated in nPLA treatment and directly inhibits apoptosis. Click here for file

Additional file 6 **EGFR1 pathway regulates apoptosis**. EGFR1 pathway regulates apoptosis and many genes from the cluster 1 and 6 (Genes that were either upregulated by MCAo or nPLA treatments) are largely found in this pathway. Click here for file

Additional file 7 **Modulation of cell death and survival pathways via HSP70**. Hspa1a (HSP70) is further upregulated in nPLA treatment than MCAo and it directly inhibits apoptosis. Click here for file

Additional file 8 **Neuronal injury mediated by glutamate on oganotypic hippocampal culture**. **(a) **Effects of concurrent dose-dependent administration of nPLA on glutamate-induced neuronal cell death. Organotypic hippocampal cultures were incubated with 0.19 mM MK801 and various concentration of nPLA [0.038 μM (0.5 μg/ml), 0.075 μM (1.0 μg/ml) and 0.15 μM (2.0 μg/ml)] separately and found to be non-toxic to the cultures. nPLA (0.038 μM, 0.075 μM and 0.015 μM) was able to protect the CA3 region from glutamate damage. Each point represents the mean ± SEM (n = 8). *: p-value < 0.01. **(b) **The effect of post-treatment of nPLA (dose-dependent) on glutamate-induced neuronal cell death. The treatment was initiated post glutamate insult (during the recovery period). The damage to CA1 and CA3 neuronal cell fields are expressed as a percentage of the area expressing fluorescence as compared with the untreated control cultures and 10 mM glutamate was taken as maximum damage. Each point represents the mean ± SEM (n = 8). *: p-value < 0.01 and #: p-value < 0.05. Click here for file
