# Supplementary figures and images for: A secretory phospholipase A2-mediated neuroprotection and anti-apoptosis
Source: BMC Neurosci. 2009 Sep 23;10:120. doi: 10.1186/1471-2202-10-120 (PMC2758888; doi:10.1186/1471-2202-10-120)

## Slide 1
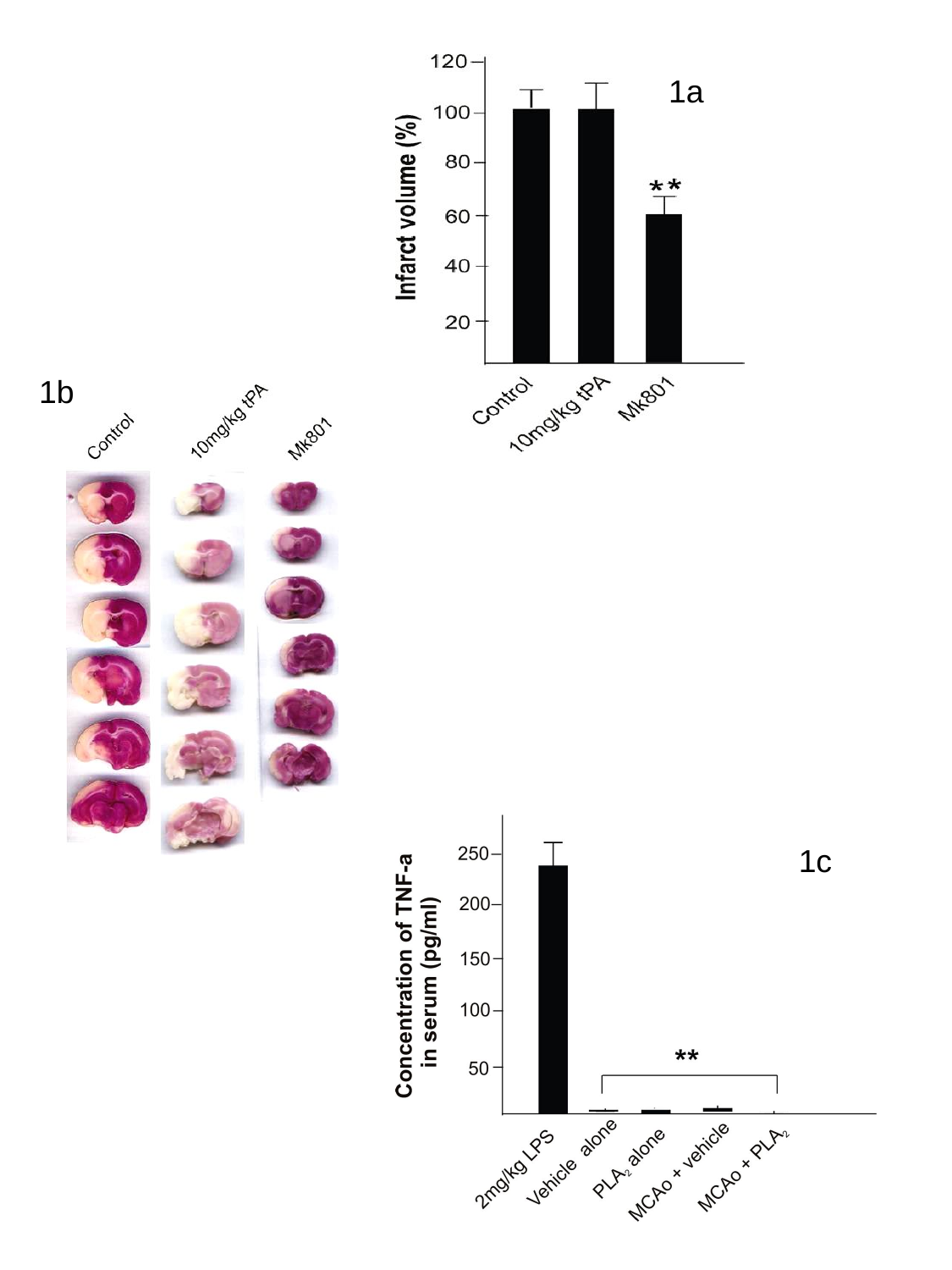

1a
1b
1c

Supplement: Additional file 1 — Rats subjected to MCAo and treated with tPA and MK801. (a) Infarct volumes were expressed as a percentage of the vehicle control ± SEM. **, p < 0.01 by unpaired Student's t-test. (b) TTC stained coronal brain sections (2 mm thick) from rats treated with tPA and MK801 (n = 6). (c) Serum TNF-α level. Rats were either treated intravenously prior to or after transient MCAo (n = 6 per treatment group). Results are the mean of duplicate experiments and expressed as concentration of serum TNF-α ± SEM. **, p < 0.01 by unpaired Student's t-test. [file 1471-2202-10-120-S1.PPT]

## Slide 1
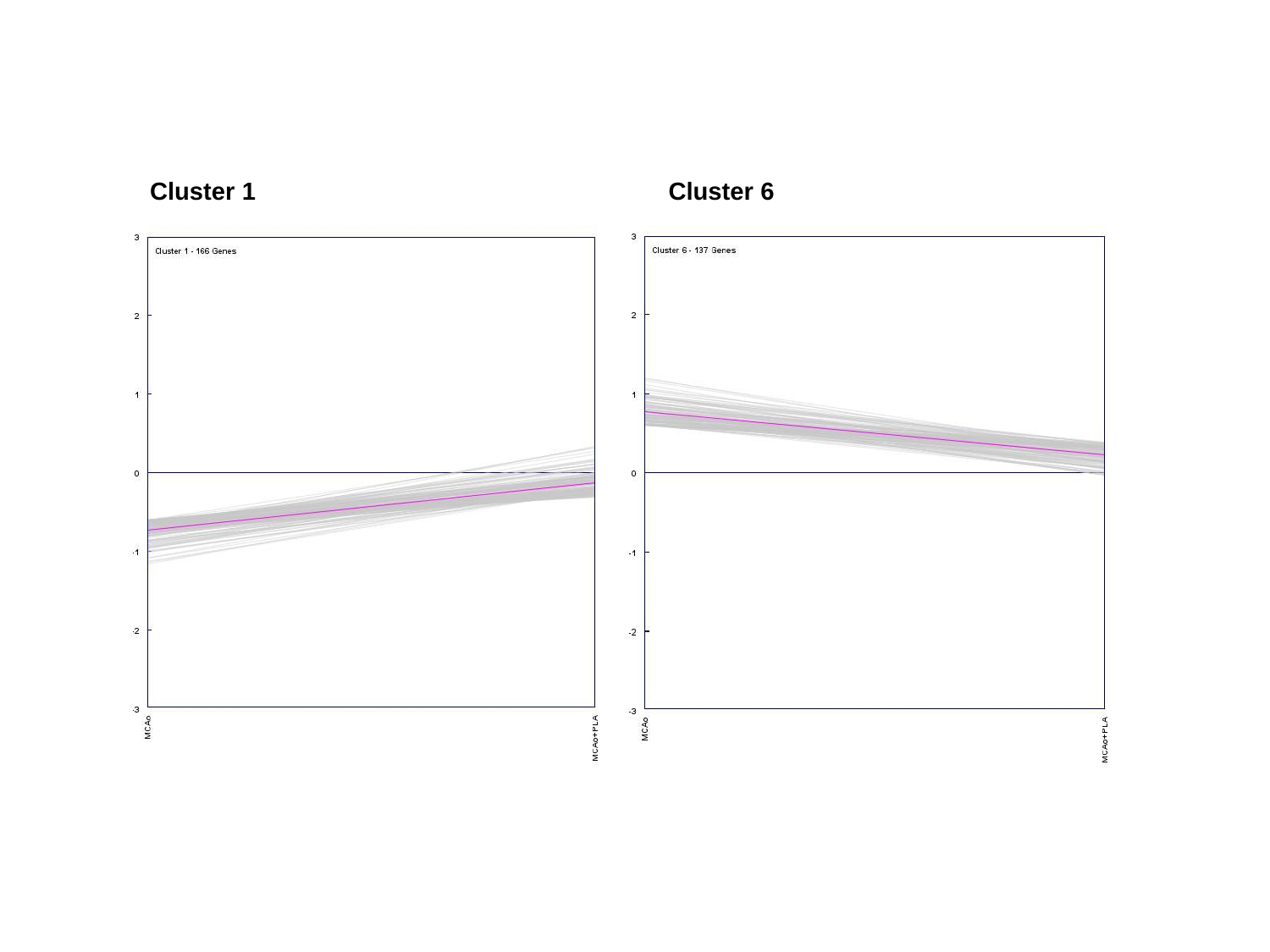

Cluster 1
Cluster 6

Supplement: Additional file 2 — Oligonucleotide microarray data analysis. The microarray dataset was clustered by K-means clustering using Genesis Software. The first data point denotes MCAo treatment and the second data point denotes MCAo+PLA treatment. Of the total 15 clusters obtained only Clusters 1 and 6 were selected. [file 1471-2202-10-120-S2.PPT]

## Slide 1
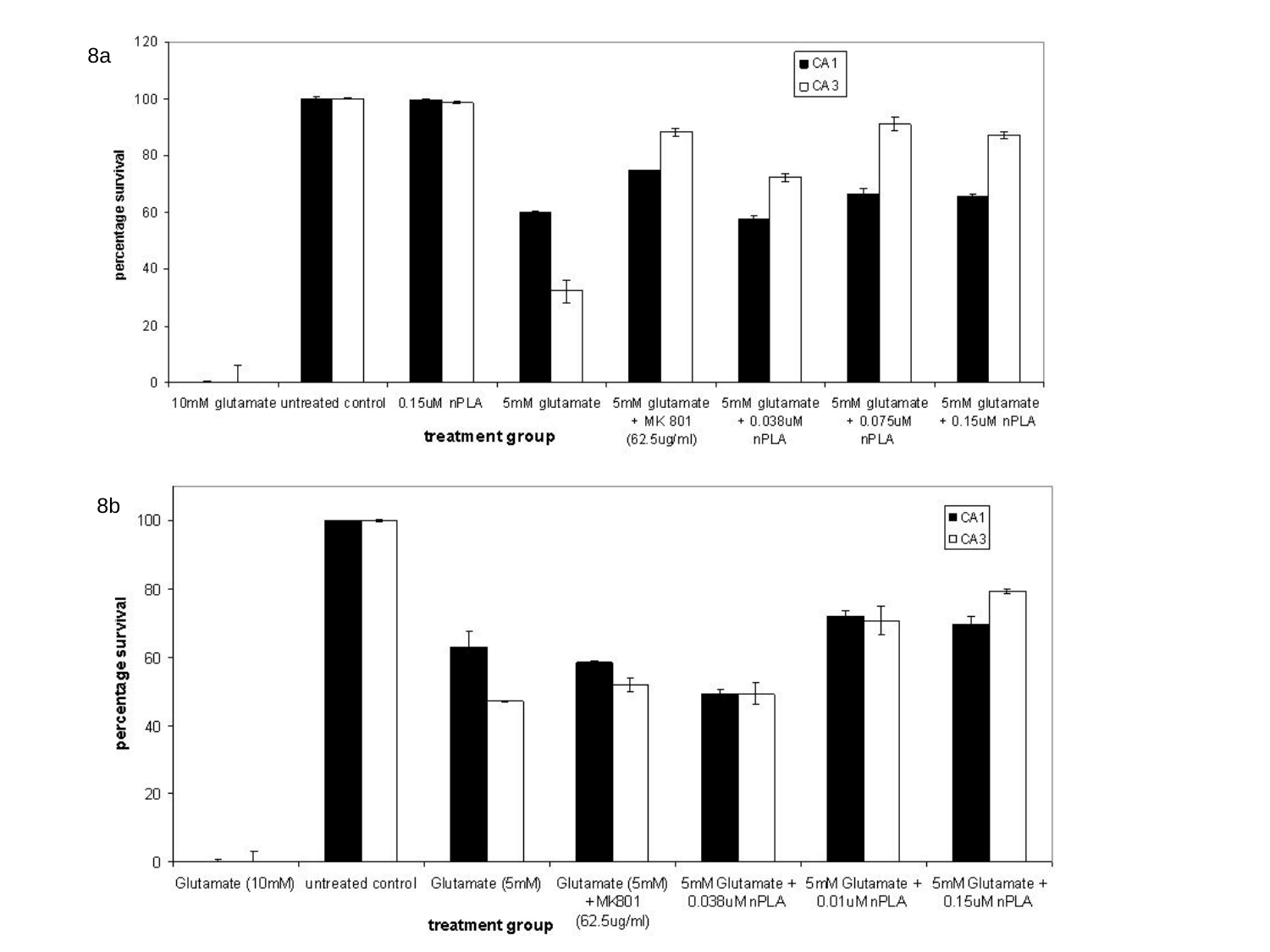

8a
8b

Supplement: Additional file 8 — Neuronal injury mediated by glutamate on oganotypic hippocampal culture. (a) Effects of concurrent dose-dependent administration of nPLA on glutamate-induced neuronal cell death. Organotypic hippocampal cultures were incubated with 0.19 mM MK801 and various concentration of nPLA [0.038 μM (0.5 μg/ml), 0.075 μM (1.0 μg/ml) and 0.15 μM (2.0 μg/ml)] separately and found to be non-toxic to the cultures. nPLA (0.038 μM, 0.075 μM and 0.015 μM) was able to protect the CA3 region from glutamate damage. Each point represents the mean ± SEM (n = 8). *: p-value < 0.01. (b) The effect of post-treatment of nPLA (dose-dependent) on glutamate-induced neuronal cell death. The treatment was initiated post glutamate insult (during the recovery period). The damage to CA1 and CA3 neuronal cell fields are expressed as a percentage of the area expressing fluorescence as compared with the untreated control cultures and 10 mM glutamate was taken as maximum damage. Each point represents the mean ± SEM (n = 8). *: p-value < 0.01 and #: p-value < 0.05. [file 1471-2202-10-120-S8.PPT]
