# Supplementary material for: A secretory phospholipase A2-mediated neuroprotection and anti-apoptosis
Source: BMC Neurosci. 2009 Sep 23;10:120. doi: 10.1186/1471-2202-10-120 (PMC2758888; doi:10.1186/1471-2202-10-120)
Supplement: Additional file 3 — NetAffex & GenMAPP analysis of microarray data. Total of 1455 genes were selected from the oligonucleotide microarray raw data based on our filtering criteria (0.6<fold change<-0.6 and detection p < 0.025). This gene list was further subjected to NetAffex analysis, K means-clustering, GenMAPP and Gene Ontology analyses. The microarray dataset was clustered by K-means clustering. Total of 15 clusters were obtained. Clusters 1 and 6 showed that the genes affected by MCAo were normalised to sham levels upon nPLA administration. [file 1471-2202-10-120-S3.DOC]

**ADDITIONAL FILE 3**

| **Cluster 1** |  |
| --- | --- |
| **MAPP Name** | **Number of gene Changed** |
| Rn_Calcium_regulation_in_cardiac_cells | 4 |
| Rn_IL-5_NetPath_17 | 3 |
| Rn_IL-7_NetPath_19 | 2 |
| Rn_Smooth_muscle_contraction | 2 |
| Rn_T-Cell-Receptor_NetPath_11 | 2 |
| Rn_B_Cell_Receptor_NetPath_12 | 2 |
| Rn_GPCRDB_Other | 2 |
| Rn_Wnt_NetPath_8 | 2 |
| Rn_G_Protein_Signaling | 2 |
| Rn_GPCRDB_Class_A_Rhodopsin-like | 2 |
| Rn_IL-6_NetPath_18 | 2 |
| Rn_IL-4_NetPath_16 | 2 |
| Rn_Androgen-Receptor_NetPath_2 | 2 |
| Rn_EGFR1_NetPath_4 | 2 |
| Rn_IL-9_NetPath_20 | 2 |

| **Cluster 6** | |
| --- | --- |
| **MAPP Name** | **Number of genes Changed** |
| Rn_Ribosomal_Proteins | 11 |
| Rn_IL-5_NetPath_17 | 4 |
| Rn_Regulation_of_Actin_Cytoskeleton_KEGG | 4 |
| Rn_Androgen-Receptor_NetPath_2 | 4 |
| Rn_Calcium_regulation_in_cardiac_cells | 4 |
| Rn_Translation_Factors | 3 |
| Rn_Smooth_muscle_contraction | 3 |
| Rn_IL-6_NetPath_18 | 3 |
| Rn_IL-4_NetPath_16 | 3 |
| Rn_EGFR1_NetPath_4 | 3 |
| Rn_Focal_adhesion_KEGG | 3 |
| Rn_Starch_and_sucrose_metabolism | 3 |
| Rn_G_Protein_Signaling | 2 |
| Rn_Inositol_phosphate_metabolism | 2 |
| Rn_IL-3_NetPath_15 | 2 |
| Rn_Nicotinate_and_nicotinamide_metabolism | 2 |
| Rn_Sphingoglycolipid_metabolism | 2 |
| Rn_MAPK_signaling_pathway_ KEGG | 2 |
| Rn_IL-2_NetPath_14 | 2 |
| Rn_Benzoate_degradation_via_CoA_ligation | 2 |
| Rn_TGF-beta-Receptor_NetPath_7 | 2 |
| Rn_Circadian_Exercise | 2 |
| Rn_TNF-alpha-NF-kB_NetPath_9 | 2 |
