# Supplementary material for: A secretory phospholipase A2-mediated neuroprotection and anti-apoptosis
Source: BMC Neurosci. 2009 Sep 23;10:120. doi: 10.1186/1471-2202-10-120 (PMC2758888; doi:10.1186/1471-2202-10-120)
Supplement: Additional file 4 — Gene Ontology analysis of genes in Cluster 1 & 6. The gene list of the clusters 1&6 were used to perform a Gene Ontology and pathway analysis using GENMAPP based on the number of genes involved in each GO term/pathway. [file 1471-2202-10-120-S4.DOC]

**ADDITIONAL FILE 4**

| **Cluster 1: Gene ontology analysis** | | | |
| --- | --- | --- | --- |
| Gene Ontology ID | GO Name | GO Type | Number Changed Local |
| 16021 | integral to membrane | Cellular component (C) | 17 |
| 16020 | membrane | C | 11 |
| 5634 | nucleus | C | 6 |
| 5783 | endoplasmic reticulum | C | 3 |
| 5576 | extracellular region | C | 2 |
| 5795 | Golgi stack | C | 2 |
| 151 | ubiquitin ligase complex | C | 2 |
| 166 | nucleotide binding | Function (F) | 9 |
| 5524 | ATP binding | F | 8 |
| 5509 | calcium ion binding | F | 7 |
| 8270 | zinc ion binding | F | 6 |
| 3824 | catalytic activity | F | 5 |
| 16787 | hydrolase activity | F | 5 |
| 4872 | receptor activity | F | 5 |
| 16740 | transferase activity | F | 5 |
| 46872 | metal ion binding | F | 4 |
| 4674 | protein serine/threonine kinase activity | F | 4 |
| 5515 | protein binding | F | 3 |
| 16491 | oxidoreductase activity | F | 3 |
| 5488 | binding | F | 2 |
| 5215 | transporter activity | F | 2 |
| 3676 | nucleic acid binding | F | 2 |
| 1584 | rhodopsin-like receptor activity | F | 2 |
| 17111 | nucleoside-triphosphatase activity | F | 2 |
| 3700 | transcription factor activity | F | 2 |
| 5525 | GTP binding | F | 2 |
| 287 | magnesium ion binding | F | 2 |
| 4842 | ubiquitin-protein ligase activity | F | 2 |
| 4984 | olfactory receptor activity | F | 2 |
| 5529 | sugar binding | F | 2 |
| 4182 | carboxypeptidase A activity | F | 2 |
| 6468 | protein amino acid phosphorylation | Process (P) | 5 |
| 8152 | metabolism | P | 4 |
| 7186 | G-protein coupled receptor protein signaling pathway | P | 4 |
| 6810 | transport | P | 3 |
| 7155 | cell adhesion | P | 3 |
| 6508 | proteolysis | P | 3 |
| 6355 | regulation of transcription\, DNA-dependent | P | 3 |
| 30154 | cell differentiation | P | 3 |
| 6886 | intracellular protein transport | P | 3 |
| 7165 | signal transduction | P | 2 |
| 6812 | cation transport | P | 2 |
| 6814 | sodium ion transport | P | 2 |
| 7283 | spermatogenesis | P | 2 |
| 6813 | potassium ion transport | P | 2 |
| 16567 | protein ubiquitination | P | 2 |
| 7049 | cell cycle | P | 2 |
| 6836 | neurotransmitter transport | P | 2 |

| **Cluster 6: Gene ontology analysis** | | | |
| --- | --- | --- | --- |
| **Gene Ontology ID** | **GO Name** | **GO Type** | **Number Changed Local** |
| 16021 | integral to membrane | Cellular Component (C) | 17 |
| 5634 | nucleus | C | 12 |
| 16020 | membrane | C | 11 |
| 5840 | ribosome | C | 8 |
| 5783 | endoplasmic reticulum | C | 5 |
| 5622 | intracellular | C | 4 |
| 5737 | cytoplasm | C | 4 |
| 5856 | cytoskeleton | C | 3 |
| 5578 | extracellular matrix (sensu Metazoa) | C | 3 |
| 5576 | extracellular region | C | 2 |
| 5643 | nuclear pore | C | 2 |
| 5813 | centrosome | C | 2 |
| 5829 | cytosol | C | 2 |
| 15935 | small ribosomal subunit | C | 2 |
| 166 | nucleotide binding | Function (F) | 16 |
| 3824 | catalytic activity | F | 12 |
| 16740 | transferase activity | F | 12 |
| 5524 | ATP binding | F | 12 |
| 3735 | structural constituent of ribosome | F | 11 |
| 5509 | calcium ion binding | F | 7 |
| 5515 | protein binding | F | 6 |
| 3676 | nucleic acid binding | F | 4 |
| 16301 | kinase activity | F | 4 |
| 5215 | transporter activity | F | 4 |
| 16787 | hydrolase activity | F | 4 |
| 4872 | receptor activity | F | 4 |
| 4674 | protein serine/threonine kinase activity | F | 4 |
| 3677 | DNA binding | F | 3 |
| 3723 | RNA binding | F | 3 |
| 8270 | zinc ion binding | F | 3 |
| 51082 | unfolded protein binding | F | 3 |
| 3700 | transcription factor activity | F | 3 |
| 5201 | extracellular matrix structural constituent | F | 3 |
| 5525 | GTP binding | F | 3 |
| 5488 | binding | F | 2 |
| 46872 | metal ion binding | F | 2 |
| 8233 | peptidase activity | F | 2 |
| 3779 | actin binding | F | 2 |
| 4713 | protein-tyrosine kinase activity | F | 2 |
| 15293 | symporter activity | F | 2 |
| 4295 | trypsin activity | F | 2 |
| 4263 | chymotrypsin activity | F | 2 |
| 4725 | protein tyrosine phosphatase activity | F | 2 |
| 287 | magnesium ion binding | F | 2 |
| 8415 | acyltransferase activity | F | 2 |
| 6412 | protein biosynthesis | Process (P) | 12 |
| 6810 | transport | P | 6 |
| 6468 | protein amino acid phosphorylation | P | 6 |
| 6457 | protein folding | P | 5 |
| 6355 | regulation of transcription\, DNA-dependent | P | 4 |
| 6350 | transcription | P | 3 |
| 7186 | G-protein coupled receptor protein signaling pathway | P | 3 |
| 6508 | proteolysis | P | 3 |
| 7264 | small GTPase mediated signal transduction | P | 3 |
| 6470 | protein amino acid dephosphorylation | P | 3 |
| 7155 | cell adhesion | P | 3 |
| 7165 | signal transduction | P | 2 |
| 7275 | development | P | 2 |
| 7242 | intracellular signaling cascade | P | 2 |
| 6811 | ion transport | P | 2 |
| 6886 | intracellular protein transport | P | 2 |
| 6334 | nucleosome assembly | P | 2 |
| 6986 | response to unfolded protein | P | 2 |
| 7243 | protein kinase cascade | P | 2 |
| 7517 | muscle development | P | 2 |
| 6817 | phosphate transport | P | 2 |
