# Supplementary material for: A secretory phospholipase A2-mediated neuroprotection and anti-apoptosis
Source: BMC Neurosci. 2009 Sep 23;10:120. doi: 10.1186/1471-2202-10-120 (PMC2758888; doi:10.1186/1471-2202-10-120)
Supplement: Additional file 5 — Inhibition of apoptosis pathway by NFκB1. NFκB1 gene is upregulated in nPLA treatment and directly inhibits apoptosis. [file 1471-2202-10-120-S5.DOC]

**ADDITIONAL FILE 5**


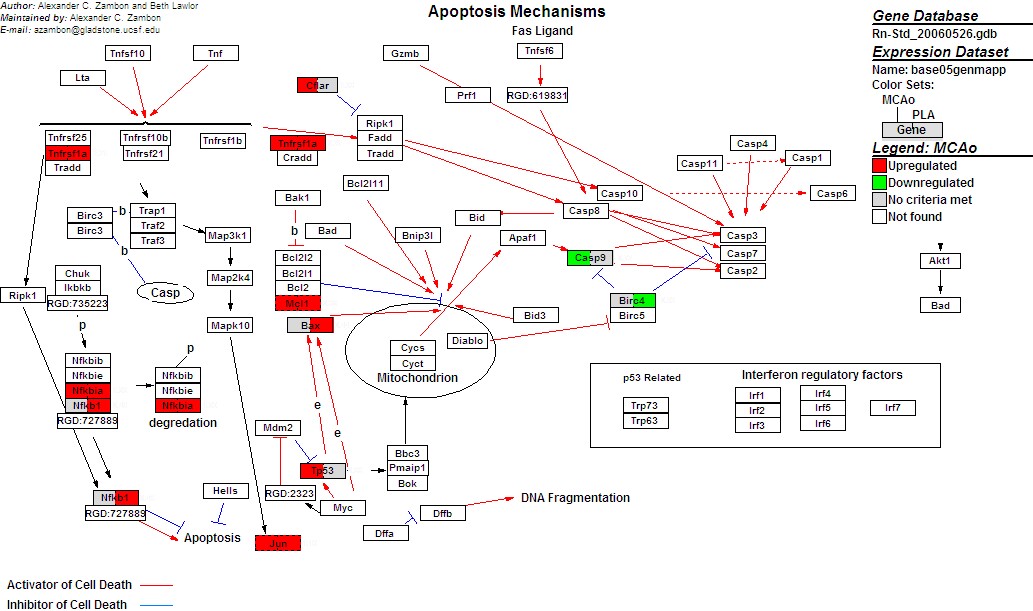


| **NFkB signaling and apoptosis pathway** | | | |
| --- | --- | --- | --- |
| **Gene name** | **Gene Symbol** | **Fold Change** | |
|  |  | MCAo | + nPLA |
| tumor necrosis factor receptor superfamily, member 1a | Tnfrsf1a | 4.63 | 3.53 |
| nuclear factor of kappa light chain gene enhancer in B-cells inhibitor, alpha | Nfkbia | 3.56 | 2.79 |
| nuclear factor of kappa light chain gene enhancer in B-cells 1, p105 | Nfkb1 | 1.38 | 1.75 |
| Jun oncogene | Jun | 2.19 | 2.10 |
| tumor protein p53 | Tp53 | 1.58 | 1.45 |
| Bcl2-associated X protein | Bax | 1.36 | 1.69 |
| myeloid cell leukemia sequence 1 | Mcl1 | 1.97 | 1.75 |
| CASP8 and FADD-like apoptosis regulator | Cflar | 2.57 | 1.43 |
| caspase 9 | Casp9 | 0.61 | 0.71 |
| baculoviral IAP repeat-containing 4 | Birc4 | 0.76 | 0.63 |
