# Supplementary material for: A secretory phospholipase A2-mediated neuroprotection and anti-apoptosis
Source: BMC Neurosci. 2009 Sep 23;10:120. doi: 10.1186/1471-2202-10-120 (PMC2758888; doi:10.1186/1471-2202-10-120)
Supplement: Additional file 6 — EGFR1 pathway regulates apoptosis. EGFR1 pathway regulates apoptosis and many genes from the cluster 1 and 6 (Genes that were either upregulated by MCAo or nPLA treatments) are largely found in this pathway. [file 1471-2202-10-120-S6.DOC]

**ADDITIONAL FILE 6**


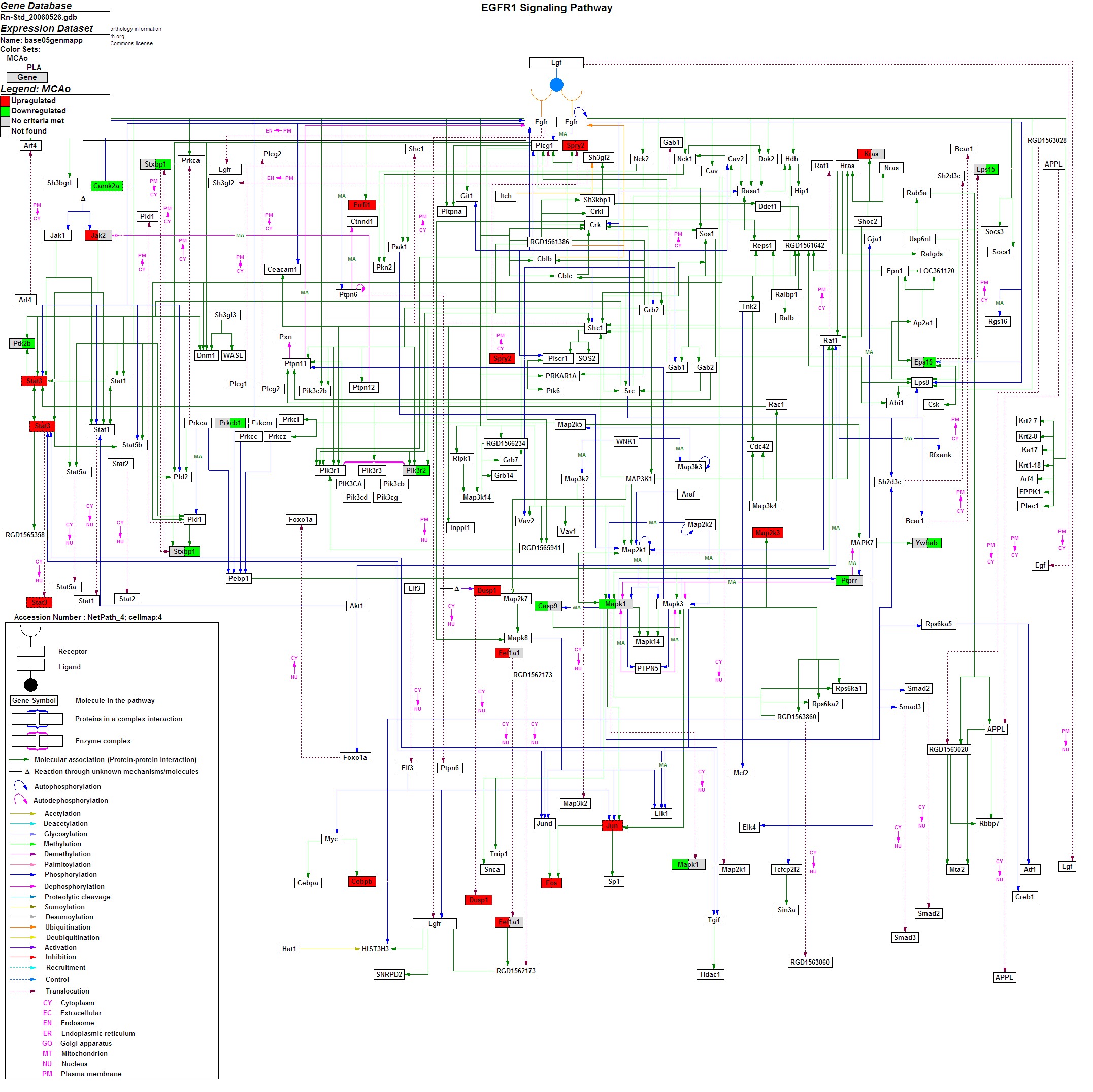


| **EGFR - Epidermal Growth factor receptor signaling pathway** | | | |
| --- | --- | --- | --- |
| **Gene Name** | **Gene Symbol** | **Fold Change** | |
|  |  | MCAo | +nPLA |
| syntaxin binding protein 1 | Stxbp1 | 0.79 | 0.30 |
| calcium/calmodulin-dependent protein kinase II, alpha | Camk2a | 0.59 | 0.64 |
| Janus kinase 2 | Jak2 | 1.72 | 1.09 |
| protein tyrosine kinase 2 beta | Ptk2b | 0.72 | 0.54 |
| signal transducer and activator of transcription 3 | Stat3 | 3.39 | 2.75 |
| protein kinase C, beta 1 | Prkcb1 | 1.06 | 0.38 |
| ERBB receptor feedback inhibitor 1 | Errfi1 | 3.34 | 3.39 |
| sprouty homolog 2 (Drosophila) | Spry2 | 1.73 | 1.91 |
| phosphatidylinositol 3-kinase, regulatory subunit, polypeptide 2 | Pik3r2 | 0.68 | 0.62 |
| CCAAT/enhancer binding protein (C/EBP), beta | Cebpb | 6.41 | 8.34 |
| dual specificity phosphatase 1 | Dusp1 | 1.78 | 2.19 |
| eukaryotic translation elongation factor 1 alpha 1 | Eef1a1 | 1.80 | 0.92 |
| FBJ murine osteosarcoma viral oncogene homolog | Fos | 22.78 | 18.38 |
| Jun oncogene | Jun | 2.19 | 2.10 |
| mitogen activated protein kinase 1 | Mapk1 | 0.62 | 1.06 |
| caspase 9 | Casp9 | 0.61 | 0.71 |
| mitogen activated protein kinase kinase 3 | Map2k3 | 2.16 | 2.33 |
| protein tyrosine phosphatase, receptor type, R | Ptprr | 0.50 | 0.71 |
| tyrosine 3-monooxygenase/tryptophan 5-monooxygenase activation protein, beta polypeptide | Ywhab | 0.97 | 0.66 |
| epidermal growth factor receptor pathway substrate 15 | Eps15 | 1.21 | 0.44 |
| v-Ki-ras2 Kirsten rat sarcoma viral oncogene homolog | Kras | 1.59 | 1.12 |
