# Supplementary material for: A secretory phospholipase A2-mediated neuroprotection and anti-apoptosis
Source: BMC Neurosci. 2009 Sep 23;10:120. doi: 10.1186/1471-2202-10-120 (PMC2758888; doi:10.1186/1471-2202-10-120)
Supplement: Additional file 7 — Modulation of cell death and survival pathways via HSP70. Hspa1a (HSP70) is further upregulated in nPLA treatment than MCAo and it directly inhibits apoptosis. [file 1471-2202-10-120-S7.DOC]

**ADDITIONAL FILE 7**


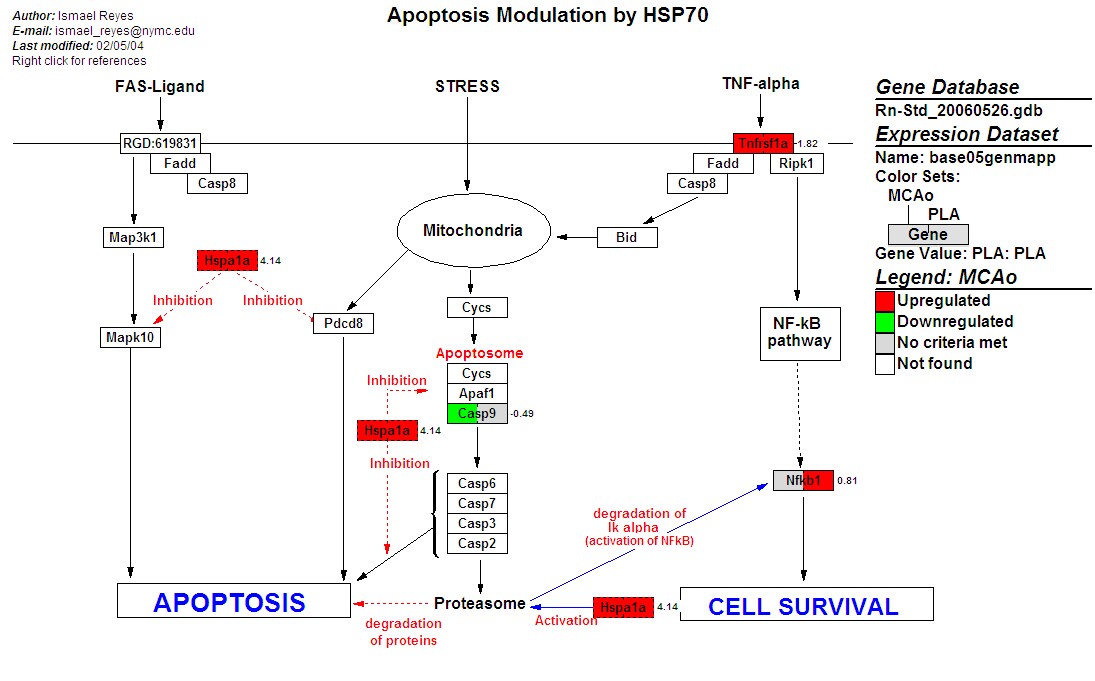


| **Apoptosis modulation by HSP 70** | | | |
| --- | --- | --- | --- |
| **Gene Name** | **Gene Symbol** | **Fold Change** | |
|  |  | MCAo | +nPLA |
| heat shock 70kD protein 1A | Hspa1a | 11.31 | 17.63 |
| nuclear factor of kappa light chain gene enhancer in B-cells 1, p105 | Nfkb1 | 1.38 | 1.75 |
| tumor necrosis factor receptor superfamily, member 1a | Tnfrsf1a | 4.63 | 3.53 |
| caspase 9 | Casp9 | 0.61 | 0.71 |
